# What we are watching—five top global infectious disease threats, 2012: a perspective from CDC’s Global Disease Detection Operations Center

**DOI:** 10.3402/ehtj.v6i0.20632

**Published:** 2013-07-03

**Authors:** Kira A. Christian, Kashef Ijaz, Scott F. Dowell, Catherine C. Chow, Rohit A. Chitale, Joseph S. Bresee, Eric Mintz, Mark A. Pallansch, Steven Wassilak, Eugene McCray, Ray R. Arthur

**Affiliations:** 1 Division of Global Disease Detection and Emergency Response, Center for Global Health, Centers for Disease Control and Prevention, Atlanta, GA, USA; 2 Influenza Division, National Center for Immunization and Respiratory Diseases, Centers for Disease Control and Prevention, Atlanta, GA, USA; 3 Division of Foodborne, Waterborne, and Enteric Diseases, National Center for Emerging and Zoonotic Infectious Diseases, Centers for Disease Control and Prevention, Atlanta, GA, USA; 4 Division of Viral Diseases, National Center for Immunization and Respiratory Diseases, Centers for Disease Control and Prevention, Atlanta, GA, USA; 5 Global Immunization Division, Center for Global Health, Centers for Disease Control and Prevention, Atlanta, GA, USA; 6 Division of Tuberculosis Elimination, National Center for HIV/AIDS, Viral Hepatitis, STD, and TB Prevention, Centers for Disease Control and Prevention, Atlanta, GA, USA

**Keywords:** epidemic intelligence, disease detection, epidemiology, global health, emergency response, CDC

## Abstract

Disease outbreaks of international public health importance continue to occur regularly; detecting and tracking significant new public health threats in countries that cannot or might not report such events to the global health community is a challenge. The Centers for Disease Control and Prevention’s (CDC) Global Disease Detection (GDD) Operations Center, established in early 2007, monitors infectious and non-infectious public health events to identify new or unexplained global public health threats and better position CDC to respond, if public health assistance is requested or required. At any one time, the GDD Operations Center actively monitors approximately 30–40 such public health threats; here we provide our perspective on five of the top global infectious disease threats that we were watching in 2012: (1) avian influenza A (H5N1), (2) cholera, (3) wild poliovirus, (4) enterovirus-71, and (5) extensively drug-resistant tuberculosis.

The spread of severe acute respiratory syndrome (SARS) in 2003 provided a stark reminder that novel pathogens could be transmitted along international travel routes with unprecedented speed (1,2). With the realization that an outbreak anywhere in the world poses a potential threat to virtually all countries (3), the US Congress in 2004 authorized the appropriation of funds to establish a global disease detection program, to be named accordingly, based at the Centers for Disease Control and Prevention (CDC), with the aim of promptly detecting and mitigating the consequences of emerging infectious diseases globally.

The Global Disease Detection (GDD) Program builds on CDC’s experience in public health surveillance, laboratory science, and outbreak prevention and control (4). The program provides a platform to develop and strengthen global capacity to rapidly detect, identify, and contain emerging infectious disease and bioterrorist threats. GDD program components include:

1. an established network of CDC public health experts stationed in GDD Regional Centers located in 10 different countries across all six World Health Organization (WHO) regions to provide ongoing technical assistance and training in various areas including field epidemiology and laboratory methods;
2. a cadre of deployable disease and refugee health experts; and
3. a centralized global events operations center dedicated to the support of two agency-wide functions: global risk- and event-based surveillance (5) and operational and financial support for a subset of CDC’s international deployments in response to events that meet specific criteria in the International Health Regulations (IHR) Annex 2 (6,7).

The Division of Global Disease Detection and Emergency Response is also the designated WHO Collaborating Center for IHR Implementation of National Surveillance and Response Capacity (6).

To address weaknesses or gaps in global public health surveillance and response capacity, the GDD Operations Center, modeled on WHO’s alert and response operations (8) and established in early 2007, serves as CDC’s platform dedicated to monitoring global public health events using event-based surveillance, which is a methodology by which reports primarily from publicly available sources and usually on the internet, are reviewed for indications of any emerging threats to public health. (5,6). The GDD Operations Center has a team of six staff and a Director with professionally diverse backgrounds, e.g., human and veterinary medicine, microbiology, and epidemiology, and is situated within dedicated space located within CDC’s Emergency Operations Center (EOC), with which we liaise both during GDD Operations Center supported international deployments of CDC teams and also when the EOC is activated to respond to an international disease event. Both official information sources, e.g., ministries of health or agriculture and WHO, as well as unofficial and unverified reports from media sources are reviewed. The latter are verified through a global network of public health professionals. Information sharing is built on trust and an understanding of how to appropriately handle information, particularly when it is not in the public domain and disclosure could harm relationships with partners. Information about disease events also comes from CDC subject matter experts in both the United States and those assigned to programs abroad. We also utilize disease-specific sources, which are particularly useful with regard to pathogens that typically are laboratory-confirmed prior to reporting (e.g., influenza, polio), and although laboratory confirmation may result in delays, such etiology-specific sources typically are rapid in reporting verified cases. We monitor outbreaks from infectious and non-infectious causes including those attributable to disasters, intoxications, and chemical, radiological, or nuclear releases. We also monitor outbreaks of unknown etiology, many of which are later determined to have an infectious cause. Outbreaks among animals may also come under surveillance for known zoonotic diseases and to assess signals that may herald emerging or re-emerging outbreaks of human disease. Regardless of the type of outbreak or public health event, increased awareness of such an occurrence is critical for rapid public health response. Finally, the GDD Operations Center’s outbreak response contingency fund provides financial support to CDC programs that makes possible a prompt response to international requests for assistance.

The GDD Operations Center monitors approximately 30–40 public health threats each day. However, we most closely watch threats of particular concern to the global public health community, and more specifically, those threats that could develop into a public health emergency of international concern to which CDC may be asked to respond bilaterally by the country experiencing the outbreak, through the Global Outbreak Alert and Response Network (GOARN), or via both routes. GOARN is a formalized mechanism by which multiple institutions are able to provide outbreak assistance that is coordinated through WHO (7,8). With this perspective, we describe five of the top global infectious disease threats that we were watching in the GDD Operations Center during 2012. The GDD Operations Center is a response-driven organization, and accordingly, we also provide information here describing to which of these threats CDC responded to between January 2007 and August 2012 in the form of deploying subject matter experts, e.g., epidemiologists and/or laboratorians at the request of a country experiencing an acute outbreak of illness (Table 1). These five top threats of 2012 were based on subjective judgment regarding future risk based on input from pertinent subject matter experts across CDC and the GDD Operations Center’s expertise in conducting early warning surveillance through monitoring of global health events, and not on an analytical algorithm or quantitative method. Factors considered for selection included high transmissibility, disease burden and severity; established or pandemic potential; disease eradication; and lack of available preventive or treatment interventions. While these five diseases were selected, there were many other noteworthy diseases such as plague, yellow fever, novel coronaviruses, that were closely monitored during 2012. The same judgment is being applied to evaluate which threats to monitor during 2013, for which a follow-up report will be written. The rationale for the selection of each for 2012 is provided below:

**Table 1:** Bilateral international deployments in response to CDC’s five top global infectious disease threats and pandemic A (H1N1) 2009, January 2007–August, 2012

Year	Disease	Countries
2007	H5N1	Cambodia, Nigeria, Pakistan
2008	Polio	Angola, Anguilla
2008	Cholera	Cameroon, Guinea-Bissau, Kenya, Zimbabwe
2009	Pandemic A (H1N1) 2009	Argentina, Australia, Chile, Costa Rica, Dominican Republic, Egypt, El Salvador, Guatemala, Kenya, Mexico*, Nicaragua, Peru, Saudi Arabia, South Africa, Ukraine
2009	Polio	Benin, Burkina Faso, Côte d’Ivoire, Guinea, Kenya, Liberia, Sierra Leone, Sudan, Tajikistan, Uganda
2009	XDR-TB	Namibia
2010	Cholera	Cameroon, Haiti, Dominican Republic
2010	Polio	People’s Republic of the Congo
2011	Enterovirus-71	Vietnam
2011	H5N1	Bangladesh
2011	Polio	Chad, Democratic Republic of the Congo, Mali
2012	Cholera	Sierra Leone*
2012	Enterovirus-71	Cambodia

* Multilateral deployment through GOARN.

## Five top infectious disease threats, 2012

### Avian influenza A (H5N1)

**Fig. 1:**
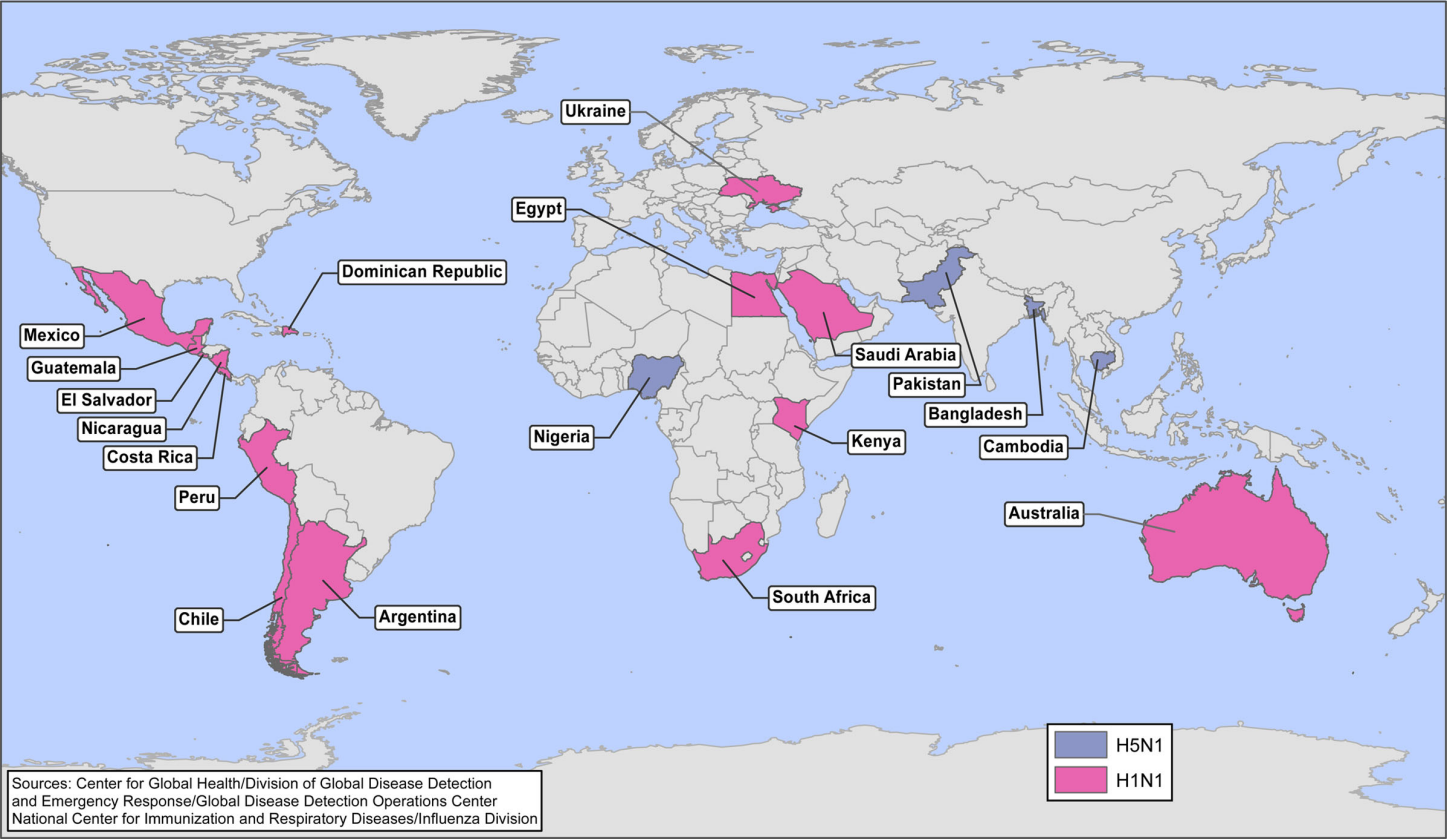
CDC’s international responses to H5N1 avian influenza and influenza H1N1-2009—January 2007–August 2012.

Avian influenza A (H5N1) was first reported to infect a human in 1997 in Hong Kong; 6 additional confirmed and 2 possible cases were reported in Hong Kong during the subsequent 7 months (9) and ultimately resulted in a total of 18 cases with 6 deaths (10). Since its emergence, this virus has been associated with continuing sporadic cases and small clusters and a high case-fatality proportion of 59% in humans. While the virus has not yet developed the capacity to spread easily from humans to humans, if it were to do so, the combination of greater transmissibility between humans, the lack of pre-existing immunity in the population, and high case-fatality proportion has the potential to cause substantial global mortality (10). Significant progress has been made worldwide over the past decade in the ability to rapidly detect and respond to the emergence of such a pathogen. While the response to the 2009 H1N1 pandemic demonstrated this growing global capacity, the potential for greater severity associated with an influenza H5N1 pandemic would be a much greater challenge. During 2012, outbreaks of highly pathogenic avian influenza H5N1 have continued to be reported in poultry, most recently confirmed in Bangladesh, Bhutan, Cambodia, Chinese Taipei, Egypt, Hong Kong, India, Japan, Republic of Korea, Myanmar, Nepal, and Vietnam (11). During 2012, 32 human infections with H5N1 influenza were reported from Bangladesh, Cambodia, China, Egypt, Indonesia, and Vietnam; most were associated with exposure to poultry, and 20 (62.5%) of these cases were fatal (12). Although influenza H5N1 remains poorly transmissible among humans, recently published research highlights the potential for mutations that would yield greater transmissibility among mammals (13–15). In addition to influenza H5N1, we continue to watch for reports of other novel influenza subtypes being reported. For example, the GDD Operations Center closely monitored pandemic A (H1N1) 2009 virus infection (2009 H1N1), which was first detected in April 2009 and spread rapidly across the world. Additionally, during 2012 we began monitoring an outbreak of highly pathogenic influenza H7N3 among poultry in Mexico first reported in June, which was subsequently associated with two non-fatal influenza infections in humans (16). Because the GDD Operations Center’s surveillance activities are solely international, we did not monitor, for example, cases of influenza A (H3N2) associated with swine in the United States; however, with our surveillance techniques we would be able to identify cases of novel influenza that occur outside the United States, such as the above example of H7N3 in Mexico. Figure 1 depicts CDC international responses to countries’ requests for assistance to cases or outbreaks of influenza H5N1 and 2009 H1N1 which occurred from January 2007 to August 2012.

### Cholera

**Fig. 2:**
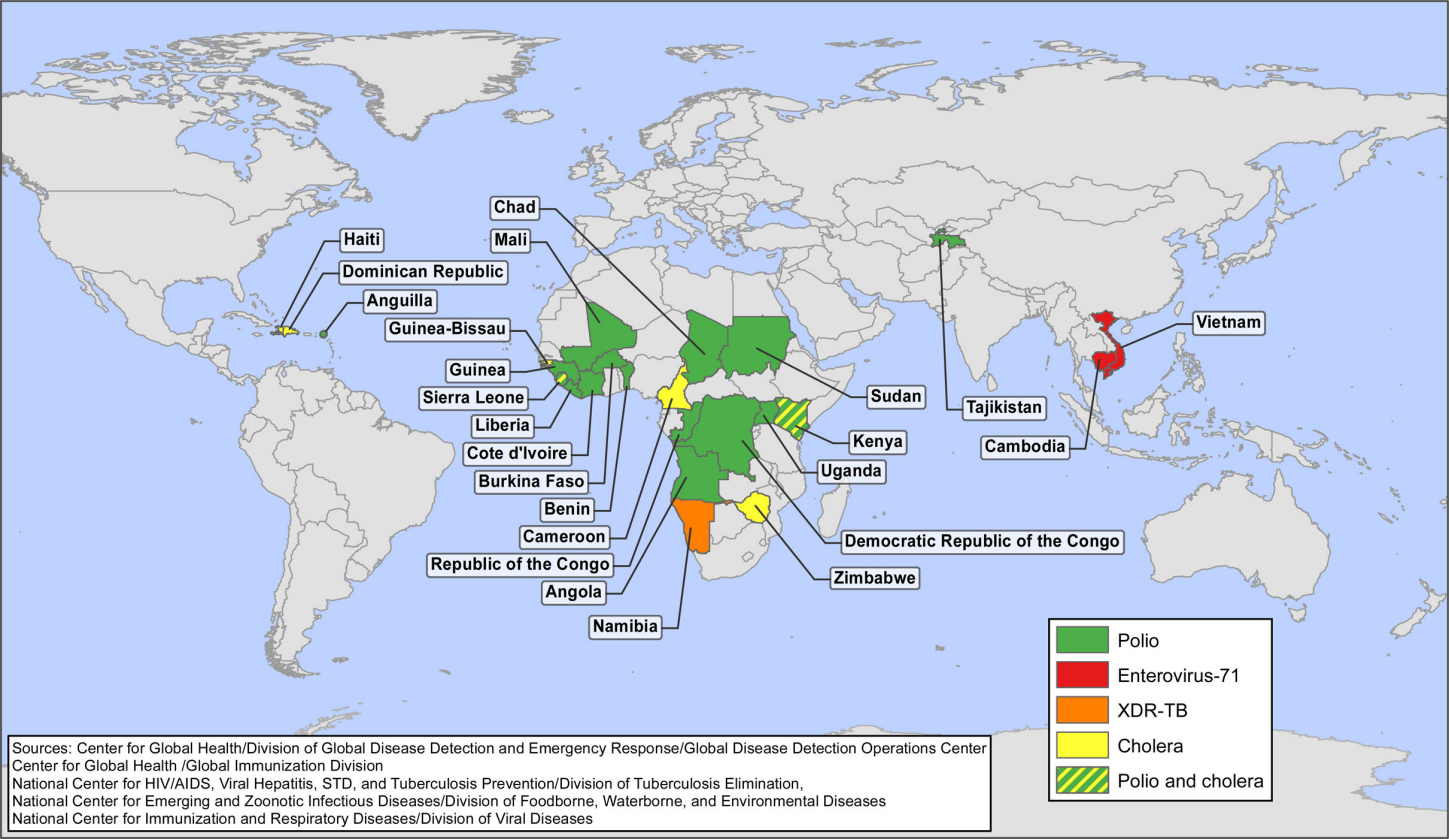
CDC’s international responses to polio, enterovirus-71, XDR-TB, and cholera—January 2007–August 2012.

Cholera warrants a place within five of the top global infectious disease threats list due to its long-established and continuing ability to infect and kill large numbers of people in a very short time. More than 100 years after the discovery of *Vibrio cholerae* and its role in human outbreaks, cholera continues to disrupt global public health. In 2011, 58 countries reported 589,854 cases of cholera and 7,816 cholera deaths to WHO (17). In 2010 and 2011, most cases were reported by Haiti, but in the previous decade, most indigenous cases of cholera reported to WHO were from sub-Saharan Africa (18). In 2009, an outbreak of over 100,000 cases occurred in Zimbabwe, and subsequently spread to neighboring South Africa and Zambia, causing thousands of additional cases (19). During 2012, the GDD Operations Center monitored outbreaks of cholera, in chronological order, in Haiti, Dominican Republic, Democratic Republic of the Congo, Somalia, Angola, Uganda, Sierra Leone, Republic of Congo, Guinea, Ghana, Mozambique, Cote d’Ivoire, Cuba, Niger, and the Philippines. In the United States and other developed countries with robust water and sanitation infrastructure, widespread outbreaks of cholera are unlikely to pose a significant threat to public health; however, cholera remains important to the global community because of its efficient transmission across vulnerable populations in countries with less well-developed infrastructure. Despite the low risk from epidemic cholera, it remains a threat in the Western Hemisphere: between January 1991 and December 1993, epidemic cholera spread throughout Latin America after first being introduced into Peru; over 1,300,000 cases and over 11,000 deaths were reported from the region between 1991 and 1996 before the epidemic ended (20). More recently, cholera has been reported from Haiti for the first time (21,22). Since the beginning of the outbreak in Haiti in October 2010 through the end of 2012, 635,980 cases and 7,912 deaths have been attributed to cholera (23). In November 2010, suspected cases of cholera were first reported from adjacent Dominican Republic, and from the first week of January 2011 to mid-December 2012, there were 28,571 cases of cholera with 418 deaths associated with this outbreak in that country (24). Cases associated with a wedding in the Dominican Republic in 2011 were reported from Venezuela, Spain, Mexico, and the United States (25), and cases among travelers returning to or coming from Haiti have been reported in the United States, Canada, Brazil, and the Bahamas (17,26). Additionally, an outbreak of cholera in July 2012 in Granma Province, Cuba attributed to the same serotype found in Haiti and the Dominican Republic: *V. cholera*, serogroup O1, serotype Ogawa, Biotype El Tor indicates potential spread from Hispaniola (27). Figure 2 depicts CDC’s international responses to outbreaks of cholera from January 2007 to August 2012.

### Poliomyelitis (polio)

Polio’s most visible current-day legacy is the permanently paralyzed victims on the streets of affected countries worldwide. In 1988, the World Health Assembly resolved to eradicate polio and as a result the global incidence of polio associated with wild polioviruses decreased from an estimated 350,000 cases in 1998 to 1,997 cases in 2006, and subsequently to 222 cases reported as of January 22, 2013 (symptom onset during 2012, reported in January 2013) (28,29). The number of countries that continue to have endemic circulation of polio has been reduced to three: Pakistan, Afghanistan, and Nigeria. Although transmission of types 1 and 3 polio continue to be reported, albeit in declining numbers, wild type 2 polio virus circulation was last reported in October 1999 (30) from Aligarh, Western Uttar Pradesh, India (31). The elimination of type 2 polio was a milestone for the Global Polio Eradication Initiative, which allowed strategies to focus on the eradication of poliovirus types 1 and 3 (30,32). In December 2011, the CDC Director activated the CDC Emergency Operations Center for the final push toward eradication. Eradicating the final 0.06% of polio is likely to be the greatest challenge. In the GDD Operations Center we monitor not only countries with endemic circulation, but also countries that report imported cases, which during 2012 was limited to Chad (28). Figure 2 shows CDC’s international responses to requests for assistances by countries experiencing cases or outbreaks of polio from January 2007 to August 2012, as reported to the GDD Operations Center. The importance of monitoring polio infections is critical now and will continue to be paramount in the post-eradication era, as even one case will represent an international public health emergency.

### Enterovirus-71

First described in 1974, this pathogen is similar to polio in its propensity to cause very severe neurologic disease. Beginning in 1997, it has caused widespread outbreaks across parts of Asia. Even though evidence of enterovirus-71 (EV-71) circulation in many other parts of the world is now being documented, with the first cases even preceding the case identified in California in 1969, the remainder of the world has only occasionally experienced the large outbreaks that have been seen in countries of Southeast Asia (33,34). Because of the lack of an effective treatment or vaccine, and because contact transmission in school and day care settings allows for efficient spread, these recent outbreaks of severe and fatal EV-71 disease across parts of Asia are a cause for concern. A notable feature of these recent outbreaks due to EV-71 is the severe and fatal disease among young children. The primary clinical manifestations include non-specific febrile illness and hand–foot–mouth disease, but approximately 2 in 10,000 children experience severe morbidity including brainstem encephalitis, pulmonary edema, and hemorrhage; to date, there is no explanation as to why some children develop more severe outcomes. Although several genetic lineages of virus are distinguished, there is no specific marker of higher pathogenicity and a range of genetic strains caused devastating outbreaks in the 2000s in Malaysia (35), China, and Taiwan (36). The fact that these strains are detected in many other parts of the world contributes to the uncertainty of why these outbreaks are more common in southeastern Asia. More recently, large outbreaks of severe hand–foot–mouth disease and fatal EV-71 have been reported from Cambodia and Vietnam. The outbreak in Cambodia was first identified in July 2012 as an outbreak of unknown etiology. Sixty-one children aged 7 years or younger presented to two different hospitals in Cambodia with high fever and neurologic and/or respiratory signs and symptoms. Of these patients, 46 died within 24 hours of admission, and the majority of the others died within 3 days. The outbreak in Vietnam began in July 2011 with a significant surge of cases being reported from the south of the country, and by the end of December 2012, there were over 148,366 cases of hand–foot–mouth disease with 45 deaths reported from 63 provinces, with cases being reported from the north of the country, indicating widespread distribution throughout the Vietnamese population (37). Both of these outbreaks are typical of the EV-71 outbreaks that have been reported from the region. Figure 2 depicts CDC’s responses to EV-71 in Vietnam (2011) and Cambodia (2012). A geographically widespread outbreak attributable to a highly transmissible pathogen like EV-71 requires close monitoring and effective response.

### Extensively drug-resistant tuberculosis

The global incidence of tuberculosis (TB) has been in a slow decline since the early 2000s. However, TB was responsible for 1.4 million deaths worldwide in 2011 (38). Additionally, the emergence and spread of multidrug-resistant (MDR) and extensively drug-resistant tuberculosis (XDR-TB), first identified in Tugela Ferry, KwaZulu-Natal, South Africa in 2005, pose a rising threat to global TB control (39). Morbidity and mortality are consistently higher among patients infected with MDR and XDR-TB, primarily because of the delays in diagnosis, limited or no options for antimicrobial therapy, complicated patient management and increased treatment costs (39). In 2009, it was reported that in the United States the cost of hospitalization for one XDR-TB patient was estimated to average $483,000 (40). According to WHO, by mid-2011, 84 countries had reported one or more cases of XDR-TB (38) and in the United States, 6 cases of XDR-TB were reported (41). In impoverished areas and vulnerable populations, the presence and spread of a demonstrably efficient human pathogen that in some situations has become almost untreatable with currently available agents warrants careful observation. In 2009 CDC responded to cases of XDR-TB in Namibia in an effort to mitigate further spread of illness (Fig. 2). Surveillance for resistant TB among global migrants and refugees is also imperative: in 2005, an outbreak of MDR-TB was identified in US-bound Hmong refugees from Thailand (42). Co-morbid conditions put vulnerable populations at further risk. Drug-susceptibility testing for first- and second-line TB drugs is unavailable in most settings with high incidence of TB, thereby creating the opportunity for emergence of XDR-TB when MDR-TB is inadequately assessed for drug susceptibility, and, treated inadequately (39). We include XDR-TB on the short list of pathogens to be monitored closely because of its potential for more widespread transmission. If XDR-TB became widespread, its severity and the difficulty of case management and infection control could cause considerable challenges for global public health.

## Summary

This perspective describes five of the top global infectious disease threats of particular concern to the CDC as a ‘snapshot’ of what we monitored during 2012 and will guide subjective judgment when determining which threats will be most closely monitored during 2013. It does not necessarily describe those diseases that CDC finds most important or those that require the most resources. Fortunately, the majority of outbreaks remain localized, and the global spread of a truly novel pathogen is rare.

## Addendum–June, 2013

### MERS-Coronavirus

Coronaviruses are a large family of viruses found in animals and humans. In both populations, coronaviruses cause a range of symptoms varying from mild, such as the common cold, to those seen in more serious respiratory illnesses in humans such as SARS. The Middle East Respiratory Syndrome Coronavirus (MERS-CoV) is a strain of coronavirus first identified in a specimen from a 60 year-old man in Saudi Arabia who developed severe respiratory disease, renal failure and died in June 2012 (43).

As of 14 June 2013, the total number of cases of MERS-CoV stands at 58 with 33 fatalities, resulting in a case-fatality proportion of 57%. These include 43 cases with 27 fatalities in KSA; two fatal cases from Jordan; two cases from Qatar; three cases with two fatalities from UK; two cases and one death from France; two cases from Tunisia; one fatal case from UAE and three cases from Italy (44). Clusters of cases have occurred in health care settings or among family contacts, but human-to- human transmission has not been sustained within the community (45).

This warrants close watching throughout 2013 because this previously-unreported coronavirus is causing severe illness in humans and the epidemiology of this pathogen remains largely undescribed.

### Avian influenza A (H7N9)

The first three cases of avian influenza A (H7N9) were reported by the China Health and Family Planning Commission to WHO on 31 March 2013 after testing negative for influenza A (H3N2), pandemic A (H1N1) 2009, and A (H5N1), as well as MERS-CoV. The cases were reported from Shanghai (2) and Anhui province (1); all three cases were severe and two patients died (46).

As of 14 June 2013, there have been 132 cases with 39 deaths attributable to avian influenza A (H7N9) reported by China to WHO (47). Cases have not been reported outside of China and to date there is no evidence of sustained human-to-human transmission. One study investigating potential sources of exposure found an epidemiologic link between confirmed cases and direct exposure to poultry or live poultry markets (48). Further, reports of incident cases have declined since the closure of live poultry markets; however, it is unclear whether this decline is attributable to market closures, warmer weather in China, or other factors (49).

Like H5N1, avian influenza A (H7N9) presents the risk that it could develop mutations that confer the ability to spread efficiently between humans. This, along with the presumed lack of pre-existing immunity among humans, could lead to a global pandemic. With this, avian influenza A (H7N9) warrants watching throughout 2013 because like MERS-CoV, the epidemiology of avian influenza H7N9 is not well understood.
